# The Time to Psychology Assessment and Provision of Psychological Interventions for Perinatal Women in the Specialist Perinatal Community Mental Health Team

**DOI:** 10.1192/bjo.2024.582

**Published:** 2024-08-01

**Authors:** Oluwaseun Ibiwoye, Snehita Joshi

**Affiliations:** Lancashire and South Cumbria NHS Foundation Trust, Chorley, United Kingdom

## Abstract

**Aims:**

According to NICE guidelines on antenatal and postnatal mental health, recommendation 1.7.3:

- To identify the proportion of women referred for psychological interventions in the perinatal period who are assessed for treatment within 2 weeks of referral.
- To identify the proportion of women referred for psychological interventions in the perinatal period who start psychological interventions within 4 weeks of assessment.
- To identify the barriers to accessing psychological interventions within 6 weeks of referral of women with mental health problem in the perinatal period.

**Methods:**

The sample cohort were perinatal women referred for psychology interventions in the central specialist perinatal community mental health team (SPCMHT) between 12^th^ June 2023 and 15^th^ November 2023. Data was collected quantitatively and qualitatively. Quantitative data was collected retrospectively from the central psychology SPCMHT database shared drive and on Rio. Qualitative data was obtained through a purposeful sampling technique. Psychologists working in the central SPCMHT were identified. Survey link with specific questions was sent to the identified participants to complete. The SPCMHT psychology pathway was reviewed to clarify the local arrangement in place for women to start psychological intervention within 6 weeks of referral.

**Results:**

25 patients were identified in total in the 6-month review period. 16 had been assessed and 9 were on the assessment waiting list. The elapsed time for assessment waitlist was between 3 and 23 weeks. Of the 16 assessments, only 2 were done within 2 weeks of referral. The time to assessment for the remaining 14 patients was between 3 and 18 weeks. Following assessment, there were 11 cases on the therapy waiting list, 4 open cases, and 1 wait and watch case. The elapsed time for the therapy waitlist was between 4 and 22 weeks following assessment. None of the open cases started therapy within 4 weeks of initial assessment. Overall, none of the 25 patients were assessed or provided psychological intervention within the recommended timeframe. The qualitative theme suggests “*understaffing*” as the major barrier to prompt service provision. It also suggests a lack of awareness of the current NICE standards timeframe for provision of psychological intervention for perinatal women. The trust SPCMHT psychology pathway has no timeframe for the start of psychological therapy following assessment.

**Conclusion:**

The audit findings suggest massive delays in service provision. The major barrier to this is limited capacity compared with the number of referrals. Hence, there is need for more resource allocation for perinatal psychology service provision.

